# *N*-Halamine Hydantoin-Containing Chitosan: Synthesis, Characterization, Thermal and Photolytic Stability Studies

**DOI:** 10.3390/molecules25163728

**Published:** 2020-08-15

**Authors:** Marta Chylińska, Halina Kaczmarek

**Affiliations:** 1 Faculty of Fine Arts, Nicolaus Copernicus University in Toruń, Henryka Sienkiewicza 30/32, 87-100 Toruń, Poland; 2 Faculty of Chemistry, Nicolaus Copernicus University in Toruń, Jurija Gagarina 7, 87-100 Toruń, Poland; halina@umk.pl

**Keywords:** chitosan, *N*-halamine, surface properties, film morphology, photostability, thermal resistance

## Abstract

Current demand for new protective materials ensuring sterility is systematically growing. The purpose of this work was the synthesis of the biocidal *N*-halamine hydantoin-containing chitosan (CS-CMH-Cl) and characterization of its properties. The functionalization of the chitosan by 5-hydantoinacetic acid substitution leads to obtaining the CS-CMH polymer, which was chlorinated in next step to transform N-H into N-Cl bonds. In this study, the possibility of forming two biocidal N-Cl bonds in hydantoin ring, grafted onto chitosan chains, was proved. The structure and stability of the prepared material was confirmed by spectroscopic (FTIR, NMR, colorimetric test) and microscopic analyses (SEM, AFM). Surface properties were investigated based on contact-angle measurements. In addition, the thermal and photochemical stability of the obtained samples were determined as functional features, determining the range of potential use. It was found that both modified chitosan polymers (CS-CMH and CS-CMH-Cl) were characterized by the smaller thermal stability and more hydrophilic and rougher surface than unmodified CS. Photooxidative degradation of the obtained materials was observed mainly on the sample surface. After irradiation, the surfaces became more hydrophilic—especially in the case of the CS-CMH-Cl—which is advantageous from the point of view of the antibacterial properties. Antibacterial tests against *S. aureus* and *E. coli* confirmed the antibacterial activities of received CS-CMH-Cl material.

## 1. Introduction

Recently, there have been great expectations related to current challenges connected with polymers of natural origin, both animal and plant [1]. Such biopolymers include polysaccharides (chitin, chitosan, proteins, alginates, cellulose, pectin) that are already used in many industries today, although still on a low scale. Their applications in medicine, food, pharmaceutical, cosmetic and packaging industries can be cited. The undoubted advantages of these renewable raw materials are availability, low production cost, biodegradability, biocompatibility, nontoxicity and occurrence in various morphologic forms (e.g., films, fibers, powders, hydrogels, microcapsules or nanoparticles/nanofibers). They can be relatively easily modified due to the presence of functional groups in the repeating units in processes such as substitution, esterification, oxidation, cross-linking. A well-known example of a chemical modification is the deacetylation of chitin, leading to an acid-soluble chitosan, which greatly expands its actual and potential applications. The biologic activity of some of them (including biocidal properties, cell recognition and interactions) must also be added to the advantages of natural polymers.

The demand for biocidal polymers is related to the prevention of action of pathogens dangerous to humans. It is particularly important to use such bioactive materials in the production of medical devices, hospital tables and operating furniture, equipment for schools and kindergartens, where hygiene is a priority and traditional agents fail (e.g., drugs resistant to microbes) or are too aggressive. In the current pandemic period, new materials with biocidal properties are of particular importance.

The antimicrobial activity of the chitosan against a variety of the pathogens including fungi, algae, and some bacteria is known [2,3,4,5]. These biocidal properties are relatively weak and strongly depend on the type of chitosan, its molecular weight, degree of deacetylation, the ratio of protonated and nonproton amino groups, temperature, pH, and the type of solvent and microorganisms. However, the efficacy of the biologic action can be improved by physical or chemical modification of this polysaccharide. It is possible by incorporating appropriate bioactive ingredients into the chitosan matrix or by the chemical bonding of substances with strong biocidal properties with macromolecules. Various compounds such as metals [6,7,8] and metal oxides [9,10] in the form of the nanoparticles, antibiotics [11,12,13] or natural oil [14] can be used to modify chitosan for this purpose. A very effective modification consists in converting the amino groups of chitosan into quaternary salts of chitosan [15,16,17,18,19,20]. Such chitosan salts are characterized by the highly effective elimination of the various types of the microorganisms. However, they often they require too long to act. The disadvantage of these materials is also the lack of the possibility to regenerate their biocidal properties.

Others interesting biocidal agents are *N*-halamines, the halogen derivatives of the nitrogen (or ammonia), which are also used to modify chitosan. *N*-Halamines have been the subject of the intensive research in recent two decades [15,21,22,23,24,25]. These compounds can significantly differ their chemical structure—they may be aliphatic, aromatic, may contain Cl, Br or I and various other substituents, which affects both physical and chemical properties as well as their biologic activity and durability [24]. It has been reported that *N*-halamine polymers exhibit strong antimicrobial activity against a broad spectrum of the pathogens dangerous for human: Gram-positive and Gram-negative bacteria, fungi as well as viruses [24,26,27,28,29,30,31]. In addition, the observed biocidal properties are strong and thus the required contact time is very short (less than five minutes) [32]. Furthermore, the advantage of these materials is the possibility of their regeneration in the halogenation process, thanks to which they can be used many times [33]. Few previous works were devoted to polymeric materials based on polystyrene derivatives chemically modified with hydantoins, which were then chlorinated [34,35,36,37].

According to our knowledge, solely scarce information on the chitosan (CS) modified by a *N*-halamine can be found in a literature [38,39,40,41,42,43]. Each of mentioned works realizes different goals and concerns separate topics. For example, the antimicrobial material was obtained by a chemical modification of chitosan with 5,5-dimetylhydantoin derivative by a ring opening reaction between 3-glycid-5,5-dimethylhydantoin and chitosan [41]. It found that the chlorinated films showed good efficacy against some bacterial species (e.g., *Escherichia coli*, *Staphylococcus aureus*). Another works described modifications of CS by substitution of 1-(hydroxymethyl)-5,5-dimethylhydantoin in amino groups [21,32,40]. The use of the obtained material for coating cotton fibers, food packaging and as a component in titanium substrates for potential orthopedic applications was proposed. The fabric made of the coated fibers indicated better crease recovery and greater the breaking strength.

The method of obtaining *N*-halamine derivatives in the above-mentioned works was based mainly on a reaction between amine groups from CS and 1-(hydroxymethyl)-5,5-dimethylhydantoin. This way, one of the nitrogen atoms is blocked and hence, it is not possible to form the *N*-halamine bond responsible for the biocidal properties of the material. Similarly in other works—various types of the polymers were successfully modified with *N*-halamines, but the reactive sites were consumed in reaction [44,45,46,47]. The biocidal activity of *N*-halamine materials are strongly associated with the total halogen content because the mechanism of the microorganisms inactivation is based on direct transfer of the oxidative halogen from material to the cell [29,36].

Our previous work was dedicated to chitosan physically modified by the addition of the *N*-halamines of different chemical structure, which exhibited antibacterial activity against two strains of bacteria: a Gram-negative bacterium *Escherichia coli* and a Gram-positive bacterium *Staphylococcus aureus* [48].

The purpose of this work was to prepare a new biocidal chitosan derivative containing covalently bonded hydantoin moieties susceptible to chlorination and characterization of the properties of the obtained material with antibacterial activity. The novelty of this work is the design of a chitosan derivative (which in the hydantoin substituent has two active groups increasing the biocidicity) not described up to now in the literature and elaboration of appropriate synthesis conditions. The planned polymer was obtained by introducing 5-hydantoinacetic acid (2,4-dioxoimidazolidine-5-acetic acid, CMH) into chitosan chains using a simple, one-pot and waste-minimizing method based on reaction of the amide bond formation. The received hydantoin-containing chitosan was then chlorinated using trichloroisocyanuric acid (TClCA). The chemical structures of the received materials were characterized by nuclear magnetic resonance (NMR) and Fourier-transform infrared spectroscopy (FTIR). Furthermore, the surface properties (hydrophilicity/hydrophobicity) and morphology of the received materials with the hydantoin and *N*-halamine side-groups were examined by contact-angle measurements, scanning electron microscopy (SEM) and atomic force microscopy (AFM). It should be pointed out the surface properties play an important role in process of colonization of bacteria. Deactivation rates against *S. aureus* and *E. coli* were tested to characterize the antibacterial activities of received materials. Moreover, the conducted studies of thermal and photochemical stability of the obtained biopolymers provided additional information for the characterization of these new materials. We would like to emphasize that such investigations have not been described in contemporary literature.

## 2. Results and Discussion

### 2.1. Preparation of the Hydantoin-Containing Chitosan—CS-CMH and N-Halamine Hydantoin-Containing Chitosan—CS-CMH-Cl

**Figure 1:**
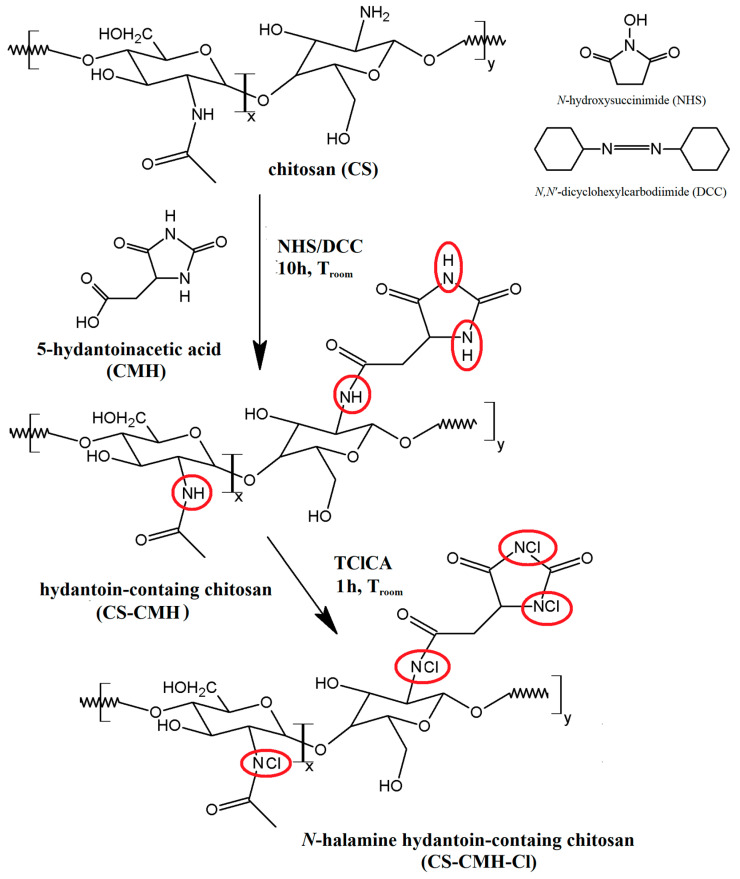
Preparation of the hydantoin-containing chitosan (CS-CMH) and *N*-halamine hydantoin-containing chitosan (CS-CMH-Cl). Active groups marked by circles.

Surface modification of biomaterials is one of the key issues in the development of the bioactive materials designed for broad medical, pharmaceutical and household applications. In this work, the chitosan was used for the synthesis of the new *N*-halamine materials containing hydantoin rings. The schematic diagram of the preparation process of CS-CMH and CS-CMH-Cl was presented in Figure 1.

The first step was the CS reaction with a 5-hydantoinacetic acid in the presence of the NHS and DCC according to the reaction of the amide bond formation (acylation). The process does not require any specific conditions—it takes place at room temperature, in an air atmosphere, in a relatively short time. During this reaction, the direct condensation of the carboxylic acid and amine was taken place. As reported in literature, the carbodiimides (e.g., DCC) are frequently used as a coupling agent in this reaction type. This method appeared an uncomplicated and useful technique for the functionalization of the chitosan, which is also confirmed by the works of other authors on similar compounds [21,49,50]. The chemical structure of the obtained polymer, confirmed by IR and NMR spectroscopies, is shown in Figure 1.

To convert hydantoin-containing chitosan film into *N*-halamine hydantoin-containing chitosan film (where N-H bonds were transformed into N-Cl bonds, which presence guarantee the biocidal properties of the obtained material), the reagents were mixed and stirred for one hour at room temperature with TClCA. Both specimens—CS-CMH and CS-CMH-Cl were obtained in the form of the films insoluble in water.

### 2.2. FTIR and NMR Characteristics of the Received Materials

**Figure 2:**
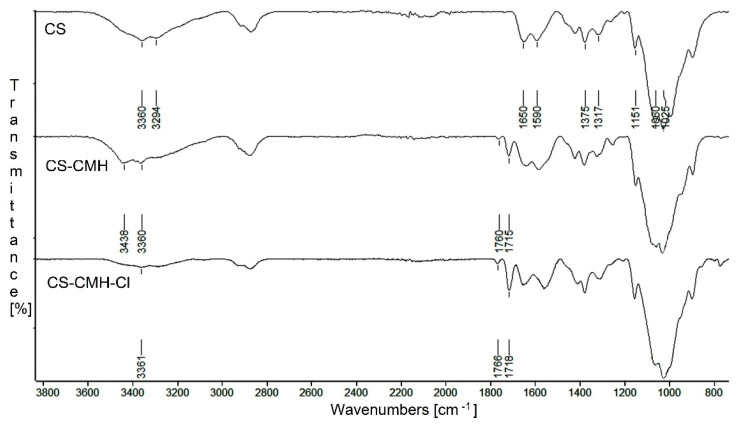
FTIR spectra of CS, CS-CMH and CS-CMH-Cl.

For confirmation of the expected structures of studied biopolymers, ^13^C NMR in solid state and FTIR spectra were done. The infrared spectra of the received chitosan films are presented in Figure 2. From the CS spectrum, the characteristic wide bands with maximum at 3360 and 3294 cm^−1^, assigned to the N-H stretching vibrations of the amine group, were found. The bands at 1650 cm^−1^ (Amide I, C=O stretching), 1590 cm^−1^ (Amide II, NH_2_ bending) and 1317 cm^−1^ (Amide III, C–N stretching) were also observed. Furthermore, the typical bands for saccharide structure appeared at 1151 cm^−1^ (anti-symmetric stretching of the C–O–C bridge), 1060 and 1025 cm^−1^ (skeletal vibration of C–O). The presence of the band at 1375 cm^−1^ attributed the -CH_3_ vibrations of the acetamide group indicates that chitosan is not completely deacetylated [51].

After chitosan modification, the new bands with maximum at 1760 and 1715 cm^−1^ appears in carbonyl region in CS-CMH spectrum. In addition, the band at 3394 cm^−1^ disappears and new band at 3438 cm^−1^ (Figure 2) is observed. This is the result of the NH groups presence from hydantoin rings. These observations confirmed the successful CMH substitution into the macrochains of the chitosan.

It can be seen, that after chlorination reaction, the wavenumbers of the carbonyl bands were negligibly shifted towards higher values (Figure 2). The wavenumbers of the C=O bands in the spectrum of the hydantoin-containing chitosan film were 1715 and 1760 cm^−1^ and in the spectrum of the *N*-halamine hydantoin-containing chitosan film—1718 and 1766 cm^−1^, respectively. The shift of the bands is related to the replacement of the hydrogen atoms with chlorine atoms, and thus, to the change of the chemical environment. Similar feature was found by Li and coworkers for the *N*-halamine modified chitosan films [41].

**Figure 3:**
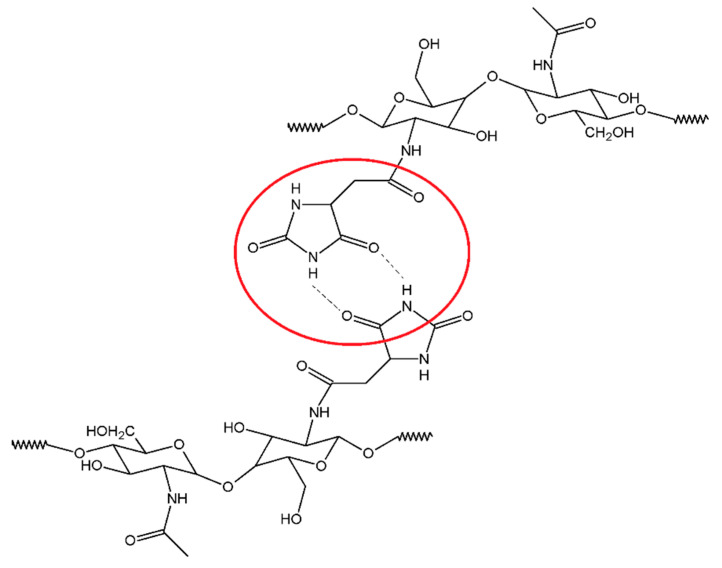
Possible interactions between CS-CMH macromolecules.

Moreover, in the CS-CMH spectrum, the characteristic bands of the stretching vibrations of the N-H bonds were observed in range 3000–3600 cm^−1^. However, in the spectrum of the CS-CMH-Cl, these bands were very week or not detected. It could be interpreted as an evidence of the high reaction yield and chlorine atom substitution. The transformation of the N-H bonds into the N-Cl bonds also causes the disappearance of the hydrogen bonds between the C=O and N-H groups (Figure 3), thus, observed shifts of the bands responsible for the stretching vibrations of the carbonyl group towards higher frequencies [52].

Both of the material components (CS and CMH) are compounds with a huge number of functional groups able to interact with each other. The tested *N*-halamines contains an electronegative nitrogen atom connected with a hydrogen atom as well as at least two carbonyl groups, hence hydrogen bonds are obvious. Such interactions compete with intramolecular hydrogen bonds in chitosan itself. A similar statement was postulated in previous works for other systems [48,53].

**Figure 4:**
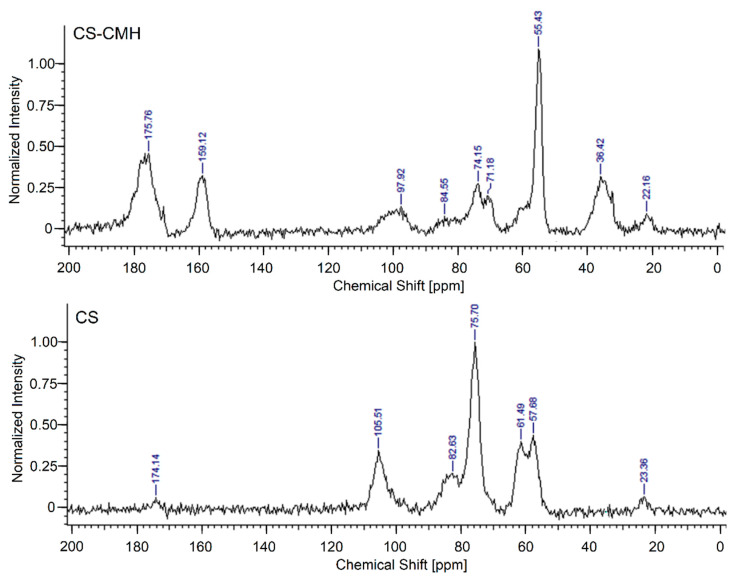
Solid-state ^13^C NMR spectra of the CS and CS-CMH.

For a more precise determination of the structures of synthesized CS-CMH, the ^13^C-NMR spectroscopy was used. The CS spectrum shows characteristic for saccharide structure peaks at around 57.7 (C_2_), 61.5 (C_6_), 75.7 (C_3,5_), 82.6 (C_4_) and 105.5 (C_1_) ppm. The very weak peaks at 23.4 (CH_3_) and 174.1 (C=O) confirmed the presence of the acetamide groups in an incompletely deacetylated chitosan (Figure 4).

In the ^13^C-NMR spectrum of the CS-CMH, the similar, characteristic peaks for saccharide structure at 55.4 (C_2,6_), 71.2, 74.2 (C_3,5_), 84.6 (C_4_), 97.9 (C_1_) ppm were also observed. However, the peaks at 36.4, 159.1 and 175.8 ppm (attributed to -CH, -CH_2_ and carbonyl groups, respectively) from substituted CMH was detected. The ^13^C NMR and IR spectra of the received chitosan films are well consistent with the proposed structures of CS-CMH (Figure 1).

The ^13^C NMR spectrum of the CS-CMH-Cl was not presented because of the negligible differences in the chemical shifts of the individual signals after chlorination. The reason for the lack of changes in the spectra of chlorinated derivative (CS-CMH-Cl) is the absence of bonds between chlorine and carbon atoms (after chlorination, the *N*-halamine bonds, i.e., N-Cl, are formed).

### 2.3. Concentration of the Free Chlorine in Aqueous Extracts

Some of the N-Cl bonds may break when the sample is soaked in water. In this case, two different forms of the chlorine are expected in the solution—free chlorine and bonded chlorine. The conventional titration method (iodometric/thiosulfate titration) permits only determinate the total amount of the chlorine. This methodology often has been used by other researchers for similar compound testing [39,41] and therefore, it is assumed that no free chlorine in the solution, which may actually be false. However, it should be pointed out that just the N–Cl bonds in modified CS structure (from which the Cl is released) are responsible for its biocidal properties. Thus, in this study, the spectrophotometric method which allows precise measurement of free chlorine released into the medium was used.

The durability of N-Cl bonds and therefore, thus, the stability of the obtained materials, was studied as the functions of the chlorine concentration in aqueous extracts. The received results were presented in Table 1.

All tested compounds were insoluble in water. This was probably the reason of the negligible dehydrochlorination of the obtained chitosan films. Thus, it can be concluded that N-Cl bonds exhibit a high stability in the aqueous environment. The concentration of the chlorine in the obtained extracts was very low (under 0.17 mg/mL). According to literature data, this is an insufficient concentration to effectively eliminate microbes [54].

### 2.4. Thermogravimetric Analysis of the Received Materials

**Figure 5:**
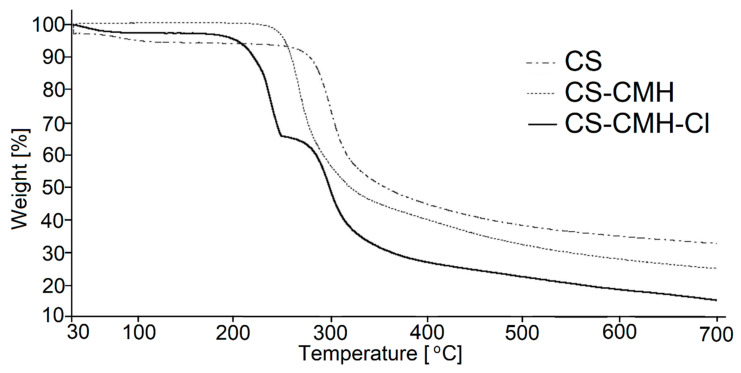
TG curves of the obtained chitosan films.

**Figure 6:**
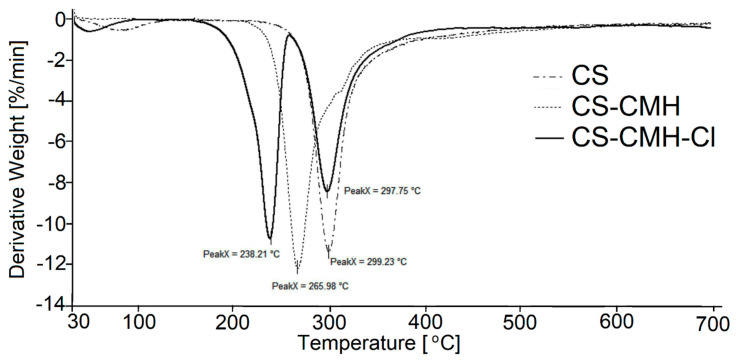
DTG curves of the obtained chitosan films.

The thermogravimetry (TG) and differential thermogravimetry (DTG) curves of the received chitosan materials, in nitrogen atmosphere, were presented in Figure 5 and Figure 6. In the first part of the thermograms for temperatures lower than 120 °C, it can be seen the weight loss by several percent, which results from the removal of water adsorbed by macromolecules.

Such effect was observed in all studied samples. The content of the water is the highest in CS (ca 5%) among studied specimens. Although, all samples were dried and stored in a vacuum dryer to a constant weight, the strongly bonded water is still present due to the high sorption capacity of the chitosan materials. These results were not included in Table 2 where summarized the thermal parameters of the TGA analyses for obtained CS materials.

The main decomposition stage of the initial CS occurs at 252 °C (maximum rate at 299 °C). The 55% mass loss in this step is associated with the degradation of the polysaccharide chains (including dehydration, deamination, deacetylation, breaking of glycoside bonds and pyranose ring opening), vaporization and elimination of the degradation products. Above 480 °C, further slow decrease of the mass loss (without any maximum at DTG) is observed up to 700 °C. TG analysis of CS is in good accordance with previous literature data [55,56,57,58]. The carbonaceous residue is about 33% at 700 °C, which is in accord with the previous report [59].

Generally speaking, CS-CMH and CS-CMH-Cl materials undergo a thermal degradation in three main steps. However, in the case of the CS-CMH, stage II and III were summarized due to difficulties in defining the border between them (shoulder peak appears). It is clearly visible that the second step of the degradation process starts earlier in the both modified chitosan than in CS. Thus, the modification of the CS leads to the decrease of its thermal stability. This is because hydantoin and *N*-halamine hydantoin groups are more susceptible to abstraction comparing to amine groups. The differences in TG curves of the unmodified CS and modified CS confirm the chemical modification of the polymer.

The TG curve of the hydantoin-containing chitosan (Figure 5) first, shows decomposition effect in the range of 237–530 °C, with a maximum degradation rate at 266 °C associated with a weight loss of 70%. This effect was attributed to a complex process corresponding to the first pyrolysis stage. The detailed analysis of the DTG curve for CS-CMH leads to a conclusion, that this step is complex and consists of at least two overlapping processes. As mentioned above, during this process, simultaneous ring dehydration, depolymerization and decomposition of the acetylated and deacetylated units of the polysaccharide occur.

It was found that the decomposition of CS-CMH-Cl started at 171 °C and a maximum degradation rate was reached at 238 °C. The thermal degradation of the polymers is initiated by breaking of the weakest chemical bond, which is N-Cl in *N*-halamine materials. Hence, the process of the chlorine abstraction from the CS-CMH-Cl should be taken to consideration as a first decomposition step. Probably the next stage in the range of 258–475 °C was oxidation by the released chlorine, accompanying the decomposition. Thus, in the presence of Cl_2_, which is oxidizing agent, chloride anions (Cl^−^) and various oxidized organic products can be generated. This can be a reason of the faster weight loss.

In consequence of chemical bonds breaking, usually free radicals are formed. The direct homolysis with formation of the chlorine atoms and macroradicals takes place upon thermal treatment. The Cl atom can recombine with any others radicals or attack the neighboring segments of the macromolecules, inducing theirs further decomposition. A macroradical with electron pair localized on the N atom in heterocyclic ring can undergo tautomerization and consequently hydrogen atom transfer from methyl or methylene groups occurs. Such transformation leads to the formation of the various products dependently on the type of the recombined radicals. The substitution of the Cl atom in the other part of the macromolecule, including backbone, is also probable.

Carbonaceous residue at 700 °C is the lowest in CS-CMH-Cl (16%) and the largest in CS (33%), while intermediate value (25%) was recorded for CS-CMH. Relatively high amount of the carbonaceous residue during process conducted in N_2_ atmosphere suggests that thermally stable crosslinking products appear resulting of the free radical recombination.

Finally, it should be pointed out that thermal analysis provided valuable information on the structure and thermal stability of the chitosan materials. However, the observed partial deterioration of the heat resistance is not a problem in the case of the predicted use of these materials at room temperature.

### 2.5. Surface Properties

The surface properties of the prepared films after each step of the modification were studied using goniometer equipped with video camera and drop shape analysis software. Quantitatively, the surface hydrophobicity/hydrophilicity was characterized by measuring the contact angle. The contact angle is defined as the angle between the substrate surface and the tangent line to the droplet surface at the point of the contact of three phases: liquid, solid substrate and gas (surrounding atmosphere). The contact-angle measurements and calculated surface free energy allow to monitor even small changes in polarity of the thin outer layer of the sample. Moreover, the calculations of dispersive and polar components of the surface free energy have brought more detailed information on the nature of the interactions and surface properties of studied samples. The increase of γ_s_^p^ is attributed to the formation of the polar groups and to the enrichment of the surface in these functional groups. The changes in γ_s_^d^ can be caused by differences of the dispersive interactions influencing the polymer density after modification. The surface free energies and their corresponding polar and dispersive components were calculated by Owens-Wendt method [60]. The received surface free energy values are given in Table 3.

The applied modification influences on the values of the total surface free energy (increase around 1–3 mJ/m^2^). It should be emphasized that these negligible changes can be detected by this method as opposed to FTIR.

It can be seen also that both CMH substitution to the CS chains and chlorination does not influence on the dispersion component values of the surface free energy. However, the results listed in Table 3 demonstrate that the modification of the chitosan by CMH and *N*-halamine CMH increases the hydrophilicity of the chitosan surface.

Presented results indicate an enhanced hydrophilicity—the increase in γ_s_^p^ was 6.26 and 5.31 mJ/m^2^ for CS-CMH and CS-CMH-Cl, respectively. Obviously, the reason for the increase in hydrophilicity is the introduction of carbonyl and amide groups into chitosan chains. There are similar reports in literature given for CS samples which were physically modified with other *N*-halamine hydantoins [36]. It was found that the values of the polar component increased even three times for CS physically modified by *N*-halamines with various structure comparing to virgin, unmodified CS.

After UV irradiation, the values of the **γ_s_** for all tested samples increased, for example for CS from 33.62 to 41.29 mJ/m^2^. The highest increase of this parameter concerns CS-CMH-Cl sample (by 9.93 mJ/m^2^). However, it can be denoted, the dispersion component values of the surface free energy for samples after irradiation were similar and correspond to unmodified CS. The differences in values of the surface free energy results from higher γ_s_^p^ values in the case of each tested samples. This is the effect of the photooxidation of the macromolecules on the sample surface. However, the photodegradation is observed only in the thin surface layer.

The photooxidative degradation of the polymers is a very complex process because different reactions can occur simultaneously. During irradiation free radicals may form and then they react with atmospheric oxygen leading to the formation of the different oxygen containing groups, namely carbonyl, hydroxyl and hydroperoxide, increasing surface hydrophilicity. The modified materials—CS-CMH and CS-CMH-Cl were more susceptible to the formation of polar groups under UV rays compared with the CS film. Probably, the introduction of the large CMH group into chitosan chain may increase the mobility of the polymer chains because the network of the hydrogen bonds is disturbed or even destroyed [61]. Presumably steric hindrance due to the presence of the hydantoin ring in the structure leads to a loosening of the structure and weaker interactions between the molecules. Increasing the elasticity and free volume between the chitosan chains is the reason for the adoption of the energetically privileged conformations. Hence, the location of the polar groups on the surface of the material is probable. Their presence will favor the adhesion of the microbial cells to this more hydrophilic surface of the material. The better contact between sample surface and pathogen cells causes enhanced biologic activity, what is required for biocidal materials. Moreover, the free volumes in polymer films facilitates the movement of the molecules or free radicals, which points to these cases the photooxidation process can be more effective.

The chlorine atoms presence could be additional reason of the high yield of the photodegradation process of the CS-CMH-Cl sample. It is possible that free, non-bonded chlorine was not removed totally and thus, this atom can act as an additional oxidizing agent.

### 2.6. SEM and AFM Analysis

**Figure 7:**
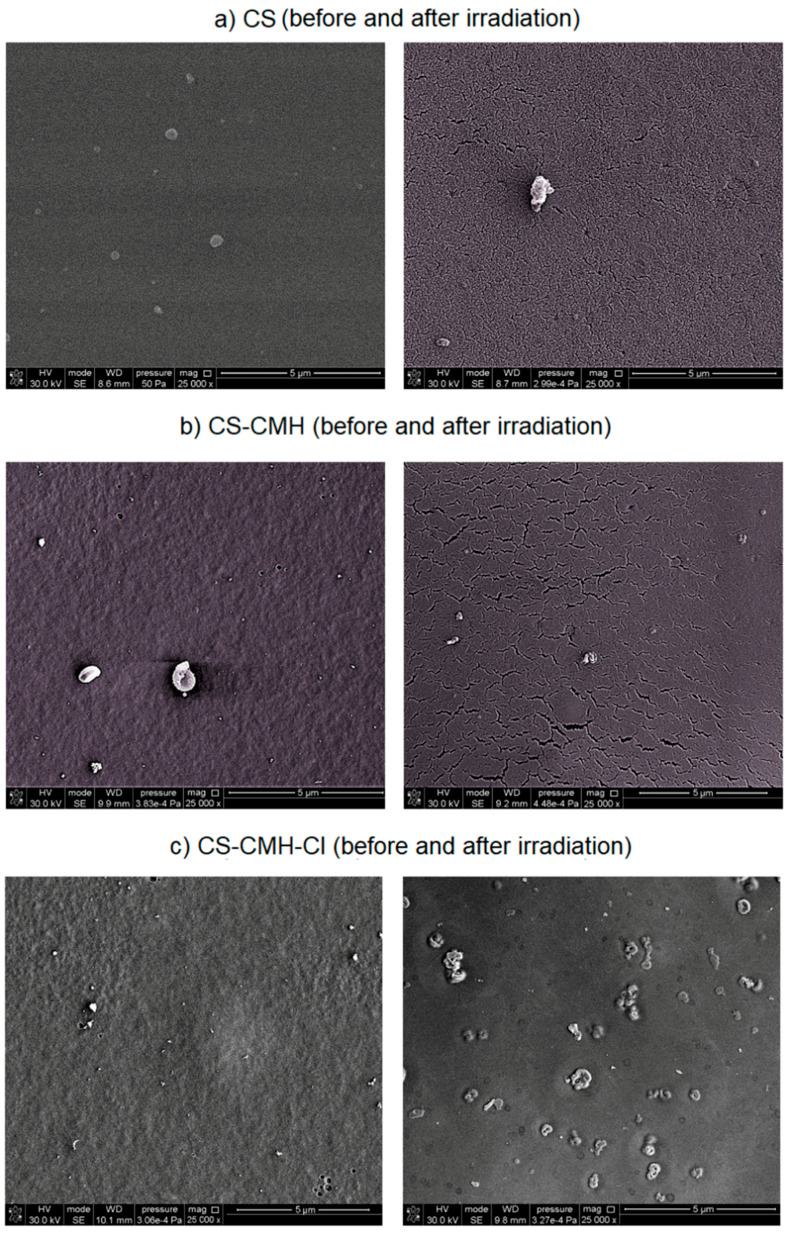
SEM images of (**a**) unmodified and (**b**,**c**) modified chitosan film surfaces before and after UV irradiation.

Modification steps altered the surface morphology of the film samples and these variations were examined by SEM analysis (Figure 7). The chitosan film exhibits a flat, homogeneous, smooth surface. However, in the case of the CS-CMH and CS-CMH-Cl films, the surfaces became relatively rough. In Figure 7 (images on the left), it can be observed that these samples exhibit a nonhomogeneous surface with some roughness caused by formation of the agglomerates. It can be associated with the chemical interactions between components of the films. It was mentioned above that, the effective interactions occur between the modified chitosan chains, especially in the case of the CS-CMH sample. The most likely process contributing to agglomeration is the formation of hydrogen bonds, as shown in Figure 3.

The SEM images of the degraded samples after one-month photoaging were presented in Figure 7 (images on the right). Surface cracks formed spontaneously during radiation exposure and the degree of the surface degradation, estimated on enlarged images, increased in modified samples. Surface cracking is usually caused by the gradient stress in exposed samples. The crosslinking of the surface material initiated by UV radiation usually causes tension stresses in the top layer, which gradually transfer to the subsequent inner layers of the sample, resulting in a stress gradient.

With increasing exposure time, the cracks propagated and intersected with each other and forming a network structure, as can be seen in Figure 7 (images in the right column).

Such a partially degraded surface with a network of microcracks will be characterized by greater liquid absorption, which in combination with increased hydrophilicity may have a positive effect on biologic activity.

Additional factors affecting the surface stress may be cyclic changes in temperature and humidity associated with the programmed operation of climatic chamber: simulated day (when the lamp is on, temperature = 30 °C) and night (dark period with the lamp off, room temperature 20 °C). Bulk samples expand and contract and these changes are accompanied by reversible water desorption/adsorption because of the high polysaccharide sensitivity to moisture. This can promote the formation of a network of small cracks in the films. Furthermore, a “wrinkled” surface of the sample exposed to radiation is observed. This effect is probably a consequence of polymer shrinkage during exposure.

In addition, in the case of the *N*-halamine hydantoin-containing chitosan, numerous crater-shaped holes were observed on the material surfaces (Figure 7c). Probably, these craters may result from the gaseous degradation products, which release from the interior of the sample. In addition, it may be the effect of bursting air bubbles trapped in the surface layer of the material [48].

These observations and conclusions were also confirmed by AFM analysis. AFM technique is common method used for surface imaging. Depending on the sample roughness, the movement of the cantilever can be very small, less than one nanometer. The analysis method based on AFM images, which gives detailed roughness maps, allows the observation of local changes in the surface topography of the tested samples. It is a complementary method to SEM analysis that does not allow the imaging of very small changes in the shape of the surface.

**Figure 8:**
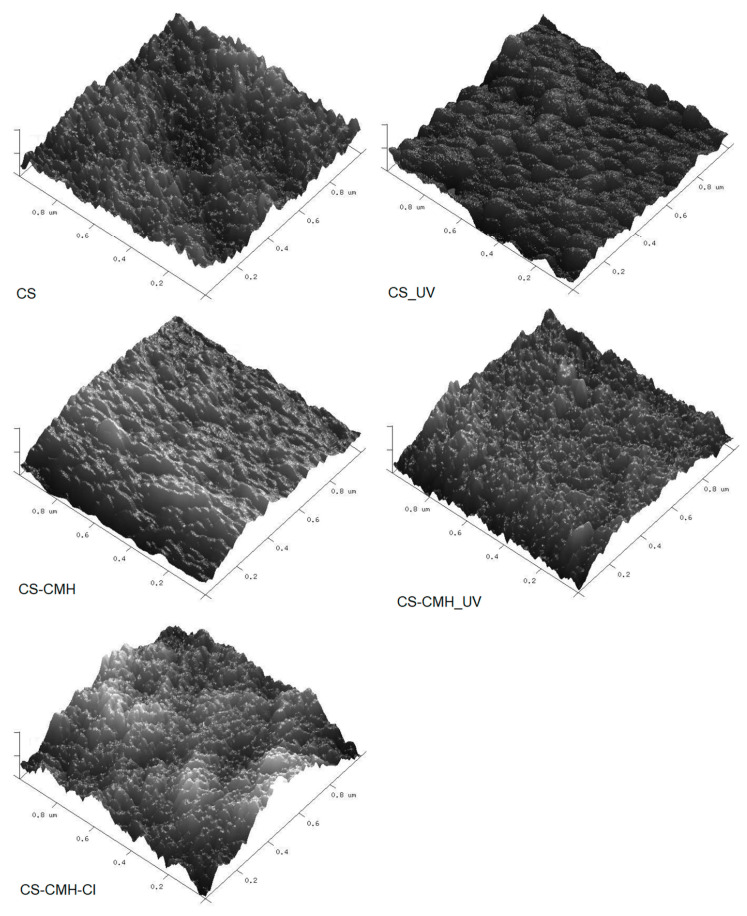
Atomic force microscopy (AFM) images of the received CS materials. Scan area: 1 μm × 1 μm and z-scale 10 nm (**left**: non-irradiated samples, **right**: irradiated samples).

AFM images and the roughness parameters (R_q_) for studied films are presented in Table 4 and Figure 8.

It can be seen that obtained images differ from each other. The chitosan film surface was flat, which was reflected in very low roughness parameter. R_q_ was equal 2.01 nm. In the cases of the modified CS samples, the film surfaces were more rugged with occurring high, wide hills, especially in the case of the CS–DMH–Cl sample. The roughness parameters for the films with the hydantoin-containing groups were higher as compared with that for the unmodified CS film. The architecture of the CS sample was more ordered than for other obtained modified samples which can be associated with the chemical reaction between carboxyl groups of the CMH and the amino groups of the chitosan. Presumably as a result of the intermolecular interactions between hydantoin-containing groups, their aggregates in chitosan films were formed.

AFM images and R_q_ parameters changed for UV-irradiated sample. After UV exposure, the morphology of the all tested film was still rough, but with the more regular architecture and less amount of the hills (Figure 8). Moreover, the surface roughness of all tested samples decreased as a result of degradation process. R_q_ values for the UV-irradiated CS and CS-CMH samples decreased when compared with that for the non-irradiated films, which may result from better ordering of the chains after irradiation.

It was mentioned above, that steric hindrance due to the presence of the hydantoin ring leads to a loosening of the structure and weaker interactions between the molecules. It could be reason of the formed holes and voids in the polymer structure, where the photoproduct can accumulate and migrate. Moreover, the CS-CMH and CS-CMH-Cl samples were more susceptible to oxidation than the CS film ones because of some chromophores already existing in these polymers before UV treatment.

The effect of UV-irradiation on morphology of the studied samples is not very clear, however, the photooxidation process could result in both formation of the low molecular weight compounds that are able to fill the valleys between hills and removal of the amorphous phase from the top surface and bonding of the polar groups, which is accordance with contact-angle measurements.

The photooxidation of the CS-CMH-Cl polymer is also enhanced due to defects arising during the film preparation. In these defects, contamination such as non-bonded chlorine is trapped which can be reason of photooxidation enhancement. Thus, irreversible, significant changes in morphology occur which was observed by AFM.

All obtained chitosan materials were characterized by irregular surface which was confirmed by SEM analysis. AFM roughness data concern only a small part of the surface; hence the results are burdened with an error. The cracks, which arise from UV-irradiation cause analysis difficult. The surface of irradiated *N*-halamine hydantoin containing sample was rough to such an extent that it was impossible to carry out AFM imaging in spite of many trials. Since the nanometric area is scanned here, the tip cannot cope with large differences in the height of points on the surface with very high roughness, thus, the image obtained is inaccurate and invalid.

### 2.7. Evaluation of Antibacterial Activity

Antibacterial effect of received materials against *S. aureus* and *E. coli* were tested. The results were listed in Table 5. It was found that CS-CMH-Cl film exhibited highly killing activities against both bacteria strains. In these cases, obtained material caused a decrease in the number of living cells by at least three orders of magnitude relative to the control samples in an hour. After 24 h, a further decrease in the number of viable cells was observed. The reason for a high efficacy in bacterium reduction is that the surface of the bacteria has a large number of hydrophilic groups such as hydroxyl and carboxyl groups which can interact with the polar groups on the surface of CS-CMH-Cl material by hydrogen bonding. This possibility allows penetrate walls of cells by chlorine atom generated from the dissociation of N-Cl bond from the *N*-halamine film [39]. *S. aureus* was more prone to necrosis than *E. coli* because its membrane is thinner and thus, easier to be broken by diffusing chlorine atoms.

## 3. Materials and Methods

### 3.1. Materials

Medium molecular weight chitosan, CS (viscosity-average molecular weight was 2.5 × 10^5^, and *N*-deacetylation degree was 85%), 5-hydantoinacetic acid, CMH, *N,N*′-dicyclohexylcarbodiimide, DCC, *N*-hydroxysuccinimide, NHS and other reagents (e.g., acetic acid, hydroxide sodium) were supplied by Sigma-Aldrich (Saint Louis, MO, USA). All reagents were pure per analysis and were used without further purification.

### 3.2. Synthesis of the Hydantoin-Containing Chitosan—CS-CMH

CS-CMH was synthesized according to the reaction of the amide bonds formation [62]. However, the details of the procedure were precisely elaborated in this work. A total of 0.5 g of CMH was first dissolved into the 20 mL 0.1-M acetic acid in 50 °C. Then, the solution was cooled to room temperature and transferred into a two-necked flask. Then, 0.4 g NHS and 0.7 g DCC (dissolved in ethanol) was added into CMH solution and stirred at 25 °C for 0.5 h. Chitosan (10 mL of 2% solution) was added subsequently. The mixture was stirred constantly for 10 h at 25 °C. After this time, the white powder was precipitated and several times rinsed by ethanol to remove the DCC and NHS residues. The final product was dried at 40 °C under vacuum for 48 h. The degree of the substitution, determined by gravimetric analysis, was ca 56%.

### 3.3. Preparation of N-Halamine Hydantoin-Containing Chitosan Film—CS-CMH-Cl

CS-CMH solution was prepared by dissolving 1.25 g of the synthesized CS-CMH powder in 25 mL of the aqueous acetic acid solution (1% *m/v*). Afterward, the solution was cast in Petri dishes and dried for 72 h at ambient conditions (T = 25 ± 2 °C) to evaporate the solvent. After air drying, the film was removed from dish, neutralized by immersing in 1-M aqueous NaOH solution at room temperature, and then repeatedly, thoroughly rinsed with deionized water. The received material was dried at 40 °C overnight.

To transform N-H bonds to *N*-halamine bonds (N-Cl), the CS-CMH film was soaked in trichloroisocyanuric acid solution (15-mmol TClCA in 10 mL of acetone) at room temperature for 1 h. After *N*-chlorination, the film was washed with deionized water thoroughly and air-dried. The washing water was tested by colorimetric test (description below, section *Concentration of Free Chlorine in Water Extracts*) to ensure that the free chlorine was completely removed. The film was dried overnight at 40 °C to achieve constant weight and stored in a desiccator at 25 °C before further tests.

### 3.4. FTIR and NMR Spectroscopy

The ATR-FTIR spectra of the obtained materials were recorded using Vertex 70v (Bruker Optics GmbH, Ettlingen, Germany) with diamond crystal and range 400–4000 cm^−1^ at room temperature. The ^13^C NMR spectra were recorded at room temperature with Bruker Avance III 700 MHz spectrometer (Bruker Optics GmbH, Ettlingen, Germany) in solid state.

### 3.5. Concentration of the Free Chlorine in Water Extracts

To study the stability of the chlorinated CS-CMH in water, the Merck reagents were used (commercially available laboratory tests). The experiment was performed using the following procedure: 0.2 g of tested film (CS, CS-CMH or CS-CMH-Cl) were immersed in distilled water (10 mL) and mixed for 15, 60 min and 24 h at room temperature. There were insoluble films on the bottom of the test tube. The extracts were removed by decantation. After sampling, free chlorine was immediately analyzed spectrophotometrically (by colorimetric test).

Free chlorine is the portion of the chlorine present in extract in the form of the dissolved elementary chlorine, as hypochlorous acid and as hypochlorite ion. This method is based on the reaction of the *N,N*-dipropyl-*p*-phenylenediamine (DPD), which forms a color semiquinoid dye with Cl_2_ in weak acidic solutions [54]. Ten milliliters of the water extract was pipetted into the test tube and vigorously shaken with DPD reagent. The pH = 5.0 was kept. The obtained mixtures, differing in intensity of pink color, allowed to stand for 1 min, and then the amount of chlorine released was determined. The absorbance at 557 nm was measured using Spectroquant^®^ Move 100 digital photometer (Merck, Darmstadt, Germany). The distilled water was used as a blank sample.

For each sample and condition, the measurements were repeated three times, and the average value was used for the evaluation. The applied method is based on standards approved for drinking water and sewage (according to U.S. EPA 330.5 and ISO: 7393–2:2017).

### 3.6. TGA Analysis

The TG, DTG and DTA curves were recorded using Simultaneous DSC-TGA analyzer STA 6000 equipped with autosampler (Perkin Elmer, Waltham, MA, USA). The following conditions were applied: temperature range: 30–700 °C, heating rate: 10 °C/min and nitrogen atmosphere. The typical thermal degradation parameters were determined from the thermogravimetric curves for all stages of the degradation: onset temperature (T_0_), temperature at maximum process rate (T_max_), temperature of the process end (T_end_), weight loss (%) and carbonaceous residue at 700 °C.

### 3.7. Surface Properties

For characterization the hydrophilicity, the contact angles (Θ) of the films were measured by the static sessile drop method with a DSA G10 goniometer (Krüss GmbH, Hamburg, Germany).

Glycerin and diiodomethane were used as the probe liquids. The contact angles were recorded immediately after dispensing 3 μL of liquid with automatic syringe onto the sample surface. The static contact angles were established using the video analysis system. The contact angle for both probe liquids was measured at ten spots. Next, the results for each sample were averaged. Measurements were performed at 25 °C under atmospheric conditions and were analyzed using the Drop Shape Analysis software, version 1.90.0.14 (Krüss GmbH, Hamburg, Germany). The statistical analysis accordance with ISO 2602:1980 standard was done for each sample. Equally important, before the measurement, all samples were conditioned at 25 ± 1 °C overnight. The surface free energy (**γ_s_**) and its polar (**γ_s_^p^**) and dispersive (**γ_s_^d^**) components were calculated from the contact angle values using Owens-Wendt method [60].

### 3.8. SEM and AFM Analyses

The SEM images of the dried chitosan films were done by a scanning electron microscope LEO 1430 working in controlled vacuum. A secondary electron (SE) detector, enabling very fine detail to be resolved, was used. Samples for SEM were sputtered with gold before imaging. AFM images of chitosan films were recorded by a MultiMode NanoScope IIIa (Veeco Metrology, Inc., Santa Barbara, CA, USA) using a silicon 186 tip (Veeco Metrology, Inc., Santa Barbara, CA, USA). The microscope was operating in a tapping mode in air and at room temperature. The images and a roughness parameter R_q_ (a root mean square) were received. The samples of CS materials before and after UV irradiation was tested.

### 3.9. Weathering Conditions

For artificial weathering, the Suntest XLS device (Atlas, Linsengericht, Germany) equipped with a xenon lamp, was used. Light was cut off by 290-nm filter made from borosilicate glass, leaving UVB (range 280–315 nm) and UVA (range 315–400 nm) rays. The following aging cycles were applied: 12 h of exposure followed by a 12-h dark period (simulation of night). The light intensity of 350 W/m^2^ was kept and black panel temperature was 35 °C. The number of the cycles was 30 (720 h, ca 1 month). The test conditions were programmed.

### 3.10. Antibacterial Test

The two strains of bacteria—*Escherichia coli* (ATCC 10536) and *Staphylococcus aureus* (NCTC 10788) were used to confirm the antibacterial properties of the received CS-CMH-Cl film. The microorganisms were incubated in nutrient broth for 24 h at 37 °C. After growing, the bacteria cells were washed by centrifugation and suspended in phosphate-buffered saline (pH was 7.2). The received bacterial suspensions were vortexed to disperse the bacterium and next, diluted to obtain appropriate concentration—10^6^–10^7^ CFU/mL. 10 mL of the bacterial culture and 0.20 g of tested material were placed into a sterile test tube. After incubation for 1 h and 24 h, the film was removed. Sample was diluted with sterile saline. 100 µL of solution was transferred to agar media and inoculated for 24 h at 37 °C. Then, the colonies were counted. The CS and CS-CMH samples were used as a reference material. Each experiment was repeated three times and the obtained results were average. 

## 4. Conclusions

Chitosan modification was designed and successfully performed to obtain material with antibacterial properties against *S. aureus* and *E. coli*. It consists of introducing *N*-halamine hydantoin groups into the polysaccharide structure. The applied preparation method based on the chemical reaction of the amide bond formation is relatively simple and inexpensive, thus, can be recommended for application in practice. Thermal stability, accelerated aging and surface properties were tested to assess the suitability of these materials as antibacterial coating. It was found that both modified chitosan polymers (CS-CMH and CS-CMH-Cl) are characterized by the smaller thermal stability than the unmodified CS due to the presence of the unstable chemical bonds. However, it will not disqualify these materials when they are used at ambient temperature or at elevated temperatures even up to 170 °C. More hydrophilic and rough surfaces were obtained after modification of the origin CS. The photooxidative degradation of the obtained materials was confirmed mainly at the sample surface. After the irradiation, the wettability of the chitosan films was changed and the surfaces became more hydrophilic, especially in the case of the CS-CMH-Cl, which is beneficial from the point of view of antibacterial properties.

## Figures and Tables

**Table 1:** Concentration of the free chlorine (mg/L) in aqueous extract of chitosan (CS) materials.

Sample	15 min in Water	60 min in Water	24 h in Water
CS	under 0.01	under 0.01	under 0.01
CS-CMH	under 0.01	under 0.01	under 0.01
CS-CMH-Cl	under 0.01	0.03	0.17

**Table 2:** Thermal parameters of the tested materials.

	Stage I				Stage II				
Sample	T_onset_ (°C)	T_max_ (°C)	T_end_ (°C)	Δ m (%)	T_onset_ (°C)	T_max_ (°C)	T_end_ (°C)	Δ m (%)	Residue at 700 °C (%)
CS	252	299	482	55	-	-	-	-	33
CS-CMH	237	266	530	70	-	-	-	-	25
CS-CMH-Cl	171	238	258	32	258	298	475	42	16

**Table 3:** Values of the surface free energy (**γ_s_**) and its polar (**γ_s_^p^**) and dispersive (**γ_s_^d^**) components for CS-samples before and after monthly irradiation.

Sample	γ_s_, mJ/m^2^	γ_s_^d^, mJ/m^2^	γ_s_^p^, mJ/m^2^
**Before Irradiation**			
CS	33.62	31.28	2.34
CS-CMH	36.43	30.17	6.26
CS-CMH-Cl	34.38	29.07	5.31
**After Monthly Irradiation**			
CS_1m_UV	41.29	29.79	11.50
CS-CMH_1m_UV	41.05	31.87	9.18
CS-CMH-Cl_1m_UV	44.31	29.45	14.86

**Table 4:** Roughness parameter, R_q_ for the obtained chitosan materials for scan area 1 μm × 1 μm, before and after irradiation.

	R_q_ (nm)	
Sample	Before Irradiation	After Monthly Irradiation
CS	2.01	1.16
CS-CMH	2.63	1.95
CS-CMH-Cl	3.73	-

**Table 5:** The antibacterial effect of obtained materials against two strains of the bacteria: *Staphylococcus aureus* and *Escherichia coli.*

Sample	Contact Time—1 h	Contact Time—24 h
***Staphylococcus aureus*** **—** **Inoculum Population: 4.1 × 10^6^ [CFU/mL]**		
CS	3.9 × 10^6^	3.1 × 10^6^
CS-CMH	4.2 × 10^6^	3.4 × 10^6^
CS-CMH-Cl	8.4 × 10^2^	1.4 × 10^2^
***Escherichia coli*** **—** **Inoculum Population: 3.4 × 10^7^ [CFU/mL]**		
CS	3.1 × 10^7^	3.4 × 10^7^
CS-CMH	3.0 × 10^7^	3.2 × 10^7^
CS-CMH-Cl	8.9 × 10^4^	7.4 × 10^4^
